# Dietary Folate Intake Is Negatively Associated with Excess Body Weight in Brazilian Graduates and Postgraduates (CUME Project)

**DOI:** 10.3390/nu11030518

**Published:** 2019-02-28

**Authors:** Gabriela A. Pereira, Josefina Bressan, Fernando Luiz P. Oliveira, Helena Maria P. Sant’Ana, Adriano M. Pimenta, Lílian L. Lopes, Helen Hermana M. Hermsdorff

**Affiliations:** 1 Department of Nutrition and Health, Universidade Federal de Viçosa (UFV), Viçosa, Minas Gerais 36570-900, Brazil; gabiamorimpereira3@gmail.com (G.A.P.); jbrm@ufv.br (J.B.); helena.santana@ufv.br (H.M.P.S.); lilian.lopes@ufv.br (L.L.L.); 2 Department of Statistics, Universidade Federal de Ouro Preto (UFOP), Ouro Preto, Minas Gerais 35400-000, Brazil; fernandoluiz@ufop.edu.br; 3 Department of Maternal and Child Nursing and Public Health of the School of Nursing, Universidade Federal de Minas Gerais (UFMG), Belo Horizonte, Minas Gerais 30130-100, Brazil; adrianomp@ufmg.br

**Keywords:** obesity, folate, B vitamins, body mass index, homocysteine

## Abstract

Folate, vitamin B6, and vitamin B12 intake can be important regulators for obesity development. Thus, we investigated the possible association between the intake of these vitamins and the excess body weight or obesity prevalence in the participants of the Cohort of Universities in Minas Gerais (CUME project). This study analyzed cross-sectional data of 2695 graduates and postgraduates from universities in the state of Minas Gerais (801 men, 1894 women, ages 36.2 ± 9.4). The first step consisted of collecting data online, and the second step consisted of blood collecting in the subsample living in the city of Viçosa and its region (Minas Gerais). Excess body weight and obesity prevalence were 38.1% and 10.1%, respectively. Inadequate intake of folate, B6, and B12 were 12, 6.3, and 11.1%, respectively. Beans/lentils and French bread presented the highest contribution to folate intake (23.45% and 10.01%, respectively). Those individuals in the third tertile for folate intake (≥511.12 μg/d) had a lower excess body weight [prevalence ratio (PR): 0.79, confidence interval (CI): 0.71–0.8] and obesity prevalence (PR: 0.60, CI: 0.45–0.78). These associations were maintained when the sample was categorized by sex. In addition, serum folate was positively associated with dietary folate (*p* for trend = 0.032) and negatively associated with serum homocysteine (*p* for trend = 0.003) in the subsample. Dietary folate intake was negatively associated with excess body weight and obesity in CUME participants, indicating the relevance of this vitamin dietary assessment.

## 1. Introduction

Obesity is a serious public health problem related to other chronic non-communicable diseases (CNCD) [1,2] in both developed and developing countries [1]. In Brazil, overweight prevalence increased by 26.3 (from 42.6% to 53.8%) and obesity reached a growth of 60% (from 11.8% to 18.9%) between 2006 and 2016 [3]. 

Obesity is a polygenic condition that interacts with external factors (e.g., physical inactivity and inadequate eating patterns) [4] and epigenetic changes (e.g., DNA methylation) [5,6]. Therefore, several dietary factors may be closely related to adipogenesis regulation through methylation modulation [4]. In this sense, folate, vitamin B6, and vitamin B12 play important roles in the DNA methylation pathway [7] because they participate in the carbon cycle with donations of methyl groups and S-adenosylmethionine (SAN) synthesis [8]. In fact, studies have shown an increase in DNA methylation by folate, vitamin B6, and vitamin B12 supplementation [9,10]. At the same time, individuals more responsive to diets for weight loss have shown higher levels of global methylation when compared to those less responsive [6]. However, most of the studies related to B vitamins intake and body composition are performed in children [5,11,12].

In this sense, we hypothesized that B-vitamins have negative associations with prevalence of excess body weight and obesity, and we assumed that a detailed approach of the food consumption in Brazilians with high degrees of instruction could generate more reliability in the findings. Due to the scarcity of studies [13,14], especially in adults, our outcomes could be helpful in the implementation of obesity prevention policies as well as subsidies for epigenetic investigations.

Overall, we investigated the association of these B-vitamins with excess body weight and obesity in participants of the Cohort of Universities in Minas Gerais (CUME project).

## 2. Materials and Methods

### 2.1. Coorte de Universidades MinEiras: Projeto CUME

The CUME project is a concurrent open cohort whose objective is to evaluate the impact of the Brazilian dietary pattern and the nutritional transition on NCD in adults ≥18 years old, graduates and postgraduates at the Universidade Federal de Viçosa (UFV), or the Universidade Federal de Minas Gerais (UFMG)—institutions located in the state of Minas Gerais, Brazil. The objective of CUME was to assess the impact of Brazilian dietary pattern and nutrition transition on the occurrence of NCD among graduates and postgraduates of federal higher education institutions located in the state of Minas Gerais, Brazil [15].

The potential volunteers were invited by e-mail and directed to the CUME’s virtual page (www.projetocume.com.br). Once they registered, they had access to the first phase of the questionnaire. The first phase contained questions about sociodemographic, anthropometric, lifestyle, and health-related issues. In this study, the following self-reported information was used sex (male/female), age (years), physical activity (yes/no), alcohol consumption (yes/no), smoking (never smoked/former smoker/currently smoke), use of vitamin supplement (yes/no) and television hours per day (>1 h/≤1 h). The second phase of the online questionnaire was sent to the volunteers after one week of the first phase response and was composed of a quantitative Food Frequency Questionnaire (FFQ) and 15 questions assessing supplement intake, cooking practices, and dietary habits.

### 2.2. Subjects

**Figure 1:**
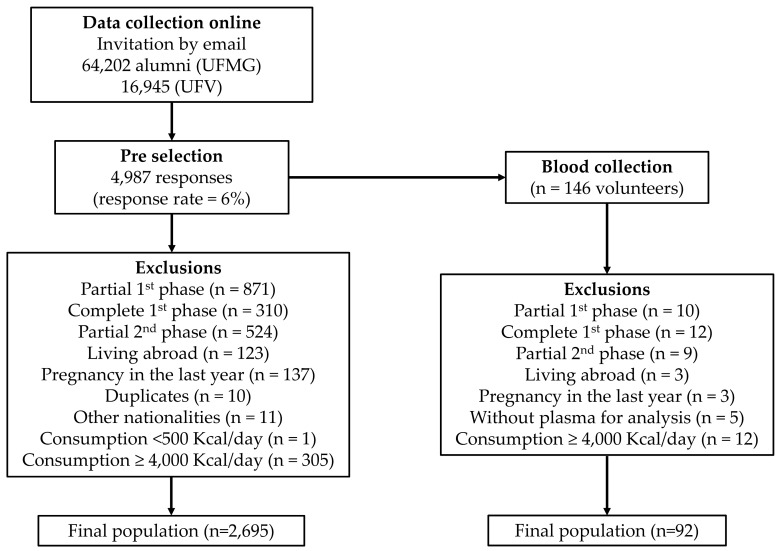
Flowchart of the study (*n* = 2695).

Of the 4986 volunteers who answered the baseline CUME questionnaire from March to August 2016, 2695 were included in the present study. The inclusion criteria adopted were: over 20 years of age, completed undergraduate or graduate courses at UFV or UFMG, Brazilian nationality and residency in Brazil over the last year, baseline questionnaire completion, women who reported not being pregnant or that they had not been pregnant in the last year, and individuals who had food consumption between 500 and 4000 kcal per day [16] (Figure 1). 

The CUME project was approved by the Human Research Ethics Committee of the educational institutions (opinion number 596.741-0/2013). All participants read the Free and Informed Consent Form and signed their consent (with an online command) before answering the questionnaire.

### 2.3. Dietary Intake Assessment

The habitual food consumption was obtained by quantitative FFQ with 144 food items previously validated for the Brazilian population [17] and some added food items that had association with NCDs. Each participant reported the frequency of consumption of a food (daily, weekly, monthly, or annually), the number of times it was consumed (zero to nine or more times), and the portion size appropriate to each food. 

Intake of macronutrients and micronutrients was calculated using information from the Brazilian Table of Food Composition [18]; in the absence of information from this, the Table of the Department of Agriculture of the United States was used [19]. The possible insufficient intake of vitamin B6 was defined as lower than 1.1 mg/day for subjects aged 50 years or less and lower than 1.3 and 1.4 mg/day for women and men aged 51 or over. For folic acid and vitamin B12, the possible insufficient intake for all ages was defined as lower than 320 μg/day and 2 μg/day, respectively [20].

### 2.4. Diagnosis of Excess Body Weight and Obesity

The weight and height self-reported in the online questionnaire were used to calculate the body mass index (BMI) as weight (kilograms) divided by height raised squared (m^2^). The self-reported weight and height were validated with a subsample of participants from the CUME project as described by Miranda et al. [21]. Adult participants were considered as having excess body weight when they presented a BMI ≥ 25 kg/m^2^; participants with BMI ≥ 30 kg/m^2^ were considered obese [22]. In turn, the elderly were considered as having excess body weight when they presented BMI ≥ 28 kg/m^2^ and obese ≥30 kg/m^2^ [23].

### 2.5. Biological Material Collection

Participants who had completed the questionnaire online and currently live in the city of Viçosa and region were invited by e-mail to participate in blood sampling (Figure 1). If the volunteer accepted, they were at the Laboratory of Energy Metabolism and Body Composition (LAMECC-DNS/UFV) in 12 h-fasting on a previously scheduled day. Blood samples were collected by a nursing professional and immediately taken to the Laboratory of Clinical Analysis (LAC-DNS/UFV), where they were centrifuged (3500 rpm, 5 °C, 15 min), aliquoted, and stored at −80 °C. The serum folic acid and homocysteine concentrations were measured from 2 mL of serum of each volunteer using the Chemiluminescence method.

A total of 146 volunteers participated in biological material collection, of which 110 had previously completed all phases of the baseline questionnaire and had their plasma collected for homocysteine and folate analysis. Of these, 92 met all the inclusion criteria and were selected for the present study (Figure 1).

The Human Research Ethics Committees of the UFV and UFMG approved the collection phase (opinion no. 1,588,799/2016), and all the participants agreed and signed a Free and Informed Consent Form regarding blood collection.

### 2.6. Statistical Analysis

The database was elaborated in SPSS^®^ software version 20.0 (SPSS Inc., Chicago, IL, USA). All statistical analyses were performed in SPSS^®^ version 20.0 and STATA^®^ version 13.0, adopting a statistical significance of 5% (α).

To control the effect of caloric intake on the relation of vitamin consumption and the occurrence of excess body weight and obesity, consumption variables were adjusted for total caloric intake by the residual method [24].

Sociodemographic characteristics among individuals with and without excess body weight were compared using the Pearson’s chi-square test. The relative contribution of each food to the daily intake of folate, vitamin B6, and vitamin B12 was obtained by calculating the ratio between each individual food nutrient content and the total nutrient amount supplied by all foods [25]. Food items were sorted in descending order according to their relative contribution averages for each nutrient [26]. Those items contributing to at least 1% of the total intake for each nutrient were presented in this study [27].

The relation between folate intake, homocysteine, and serum folate was established using multiple linear regression models adjusted for sex and age.

To assess the association between B vitamins and excessive weight, participants were categorized according to vitamin consumption tertiles. Poisson regression was used to estimate the prevalence ratio (PR) and its 95% confidence interval (CI, 95%) of excess body weight and obesity in the total sample, as well as for the male group and for excess body weight in the female group, using the first tertile of consumption of vitamins as a reference. As the obesity prevalence in the female sample was less than 10%, the odds ratio (OR) was estimated using logistic regression with the first vitamin consumption tertile as a reference.

## 3. Results

Of the 2695 volunteers who participated in this study, 70.3% were females and age mean was 36.2 (±9.4). Excess body weight prevalence was 38.1% in our sample, in which 10.1% were obese and 28% were overweight. Women and men were 22.8% and 40.6% overweight and 8.7% and 13.2% obesity, respectively. In addition, participants with excess body weight were predominantly aged ≥40 years, males, married, smokers and ex-smokers, alcohol drinkers, and watchers of television (>1 h/d) compared to participants without excess body weight (Table 1).

### 3.1. Consumption of B Vitamins and Contributing Foods

In relation to the consumption of B vitamins, 12% of the participants presented a possible insufficient intake for folate, 6.3% for vitamin B6, and 11.1% for vitamin B12, according to their Estimate Average Requirement (EAR) (IOM, 2002). Of the total food, 23 foods contributed to folate consumption, 29 for vitamin B6 consumption, and 20 for vitamin B12. In addition, beans/lentils (23.45%), French bread (10.01%), and papaya (3.93%) mostly contributed to folate consumption (Table 2), while banana (11.08%), pork (5.14%), and beef (4.67%) mostly contributed to B6 consumption, and cheese (13.98%), beef (11.97%), and fish (8.03%) to vitamin B12.

### 3.2. Folate Intake and Excess Body Weigh

Interestingly, participants included in the second and third tertiles of folate consumption intake had lower excess body weight and obesity occurrences regardless of confounding factors. The participants in the last tertile of vitamin B6 intake also had lower excess body weight or obesity in the adjusted model by sex and age. However, this association lost significance when adjusting for the other variables such as sex, age, alcohol consumption, physical activity, smoking, television hours per day, use of vitamin supplement, and daily caloric intake (Table 3). Furthermore, when categorizing the sample by gender, the associations remained significant for excess body weight and obesity. The participants in the second and third tertiles of folate consumption had lower excess body weight or obesity (Table 4). These associations did not lose significance when adjusted for age (Model 1), physical activity, smoking, television hours per day, use of vitamin supplement, and daily caloric intake (Model 2).

### 3.3. Serum Folate, Dietary Folate and Homocysteine in Subsample

**Figure 2:**
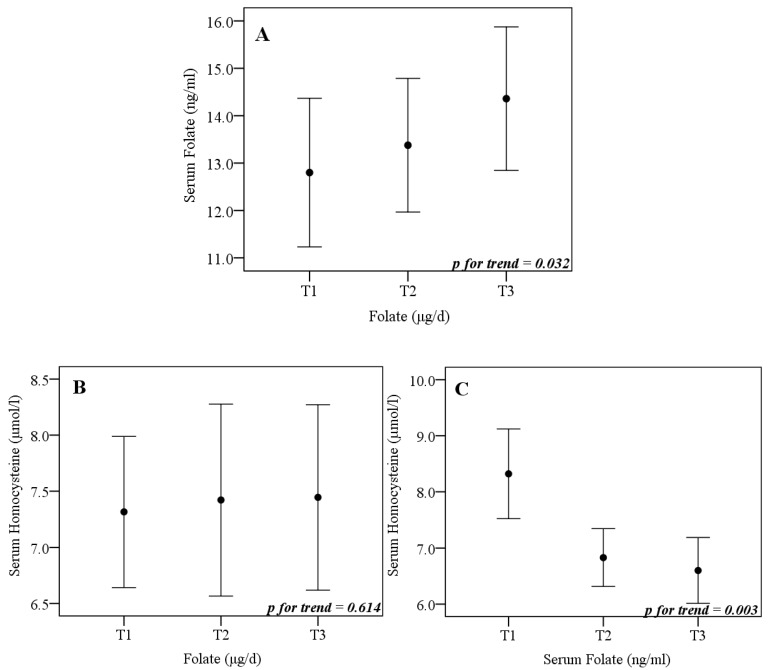
Serum folate and homocysteine values (**A** and **B**) according to tertiles of folate intake (*n* = 92), and homocysteine values (**C**) according to tertiles of serum folate in participants of the face-to-face collect of the CUME project (*n* = 110). *p* for trend in the multiple linear regression model adjusted for sex (male or female) and age (years). A and B: T1 < 414.64; T2 414.64–524.22; T3 > 524.22 µg/d. C: T1 < 11.32; T2 11.32–15.17; T3 > 15.17 ng/mL.

In the subsample participants, serum folate was positively associated with a dietary folate (*p* trend = 0.032) and negatively associated with lower serum homocysteine concentrations (*p* of trend = 0.003) regardless of sex and age (Figure 2). 

## 4. Discussion

In the present study, we observed an inverse association between folate intake and the prevalence of excess body weight and obesity. Our findings are corroborated with a previous cross-sectional study with 3767 participants from the NHANES program (National Health and Nutrition Examination Survey). This study found a trend of decreased folate intake with increased BMI [13]. Another study had shown a negative association of dietary folate with BMI [5,11] and waist circumference [12] in children as well as negative association between serum folate and BMI in adults [13,28]. The mechanisms that explain the relation between folate metabolism and obesity are still limited [13]. Obesity and related disorders have been associated with epigenetic changes such as aberrant DNA methylation patterns for genes involved in metabolic regulation and global DNA hypomethylation [1,4,6]. Studies have shown significant associations between serum folate, DNA methylation, BMI, and body fat percentage [29], and a higher folate intake from fortified foods with higher DNA methylation measured by LINE 1 in white cells [30]. Altogether, these findings support the hypothesis that higher folate intake could act as a protective factor against obesity by epigenetic mechanisms. 

In this study, serum folate was negatively associated with serum homocysteine and positively associated with dietary folate, although no significant association between serum homocysteine and folate intake was found. Homocysteine, an intermediate in the one carbon pathway, is synthesized from methionine having, as main substrates, the SAM and S-adenosyl homocysteine (SAH). Since homocysteine and SAH interconvert by hydrolysis reaction, an increase in the serum concentrations may lead to the inhibition of enzyme DNA methyltransferase, which acts by transferring groups of a one carbon from SAM to DNA [8]. In fact, increased homocysteine concentrations are inversely related to DNA methylation, while dietary folate appears to be an essential nutrient for regulating homocysteine concentrations, acting in their re-methylation to methionine [7].

Moreover, we found no association between BMI and vitamin B6 and B12 intake. Studies with children have presented similar results. Braun et al. [5] observed that the highest tertile of vitamin B6 intake was not associated with lower BMI in 2922 children (mean age 12.9 years). Unexpectedly, this study noticed higher BMI values for a greater tertile of B12 consumption, which the researchers assessed to be due to the food matrix in which this vitamin is inserted, since vitamin B12 occurs naturally in products of animal origin, which are high in protein and fat and consequently have higher caloric density [5]. Gunante et al., when investigating 1131 children enrolled in NHANES, also did not perceive a significant association between vitamin B6 intake and adiposity development [11]. In turn, a study of 128 Mexican adults, ages between 18 and 60, found no significant difference in B6 and B12 intake between individuals with and without obesity [31]. 

Another result that deserves attention was the possible insufficient intake of 12% for folate, 6.3% for vitamin B6, and 11.2% for B12 in our sample. A study with 357 individuals living in the urban area of the city of São Paulo observed inadequacy in folate consumption of 6% and 38% for men and women, respectively [32]. Data from Brazilian Family Budget Survey (POF 2008–2009) had presented inadequacy in vitamin B12 intake of 8.5% for men and 12.5% for women, for vitamin B6 of 17% and 31.9% for men and women (aged 20–50 years), respectively [33]. We believe that the low prevalence of insufficient consumption of our study may have been because the population studied was academically educated, and people with higher education tend to have lower rates of inadequacy in vitamin intake [34].

Beans/lentils followed by French Bread stood out as the foods that contributed the most to folate intake, as was found in previous studies [32,35]. Despite the mandatory fortification with folic acid established by the government for wheat flour and corn [36], beans and lentils—along with other food items such as papaya, avocado, and banana—presented an important contribution in our study to folate consumption, thus indicating that folate of natural origin presents a strong participant in the ingestion of this vitamin.

The present study has limitations. First, we did not separately evaluate B-complex vitamins from fortified and naturally occurring food sources [37], although other studies have also applied a similar methodology to estimate these nutrients [12,38,39]. Second, the lack of information about DNA methylation in our population did not allow us to confirm our hypothesis that folate intake was associated with excess body weight and obesity by epigenetic mechanisms, but this analysis is predicted for future studies in the CUME project. Lastly, the use of BMI as a measure of adiposity can be limiting because this index does not establish information on the amount and location of body fat. Nevertheless, BMI is considered a good indicator of adiposity [40,41].

The quantitative FFQ validated for the Brazilian population is a strength in our study [17] since a greater consistency is guaranteed in the data obtained. In addition, the academically educated population allowed a deepening of the questionnaire questions and greater reliability in the answers. Also noteworthy is that studies with a highly educated population have shown reliable and valid results in addition to presenting greater adherence and maintenance of the population in the study [42].

## 5. Conclusions

In conclusion, higher folate intake was negatively associated with excess body weight in Brazilian graduates and postgraduates from the CUME project, and beans and lentils are important sources of this nutrient in this population. Individual and populational strategies of nutritional education must be promoted to incentivize food-group consumption, which has reduced in these last decades in Brazil. In addition, future studies are necessary to investigate the epigenetic mechanisms (e.g., DNA methylation) involved in the relationship between dietary folate and obesity.

## Figures and Tables

**Table 1:** Socio-demographic characteristics according to the presence or not of excess body weight ^†^ of the CUME project participants (*n* = 2695).

Characteristics [*n* (%)]	Without Excess Body Weight	With Excess Body Weight	*p* Value *
	*n* = 1667	*n* = 1028	
Age			
<40 years	1278 (76.7)	644 (62.6)	<0.001
≥40 years	389 (23.3)	384 (37.4)	
Sex			
Male	370 (22.2)	431 (41.9)	<0.001
Female	1297 (77.8)	597 (58.1)	
Civil status			
Married or stable union	775 (46.5)	557 (54.2)	<0.001
Single, separated/divorced, widower	892 (53.5)	471 (45.8)	
Practice of physical activity			
Yes	1306 (78.3)	744 (72.4)	<0.001
No	361 (21.7)	284 (27.6)	
Consumption of alcoholic beverage			
Yes	1207 (72.4)	781 (76.0)	0.041
No	460 (27.6)	247 (24.0)	
Use of vitamin supplement			
Yes	496 (30.0)	258 (25.3)	0.009
No	1155 (70.0)	760 (74.7)	
Smoking			
Yes	292 (17.5)	252 (24.5)	<0.001
No	1375 (82.5)	776 (75.5)	
TV time per day			
≤1 h	1015 (60.9)	513 (49.9)	<0.001
>1 h	651 (39.1)	515 (50.1)	

* *p* values using Pearson’s chi-square test. ^†^ Excess body weight: BMI ≥25 kg/m^2^ (WHO, 1998) e BMI ≥ 28 kg/m^2^ (OPAS, 2002) for adults and elderly respectively. Legend: BMI: body mass index.

**Table 2:** Contribution (%) of food items to the dietary folate, vitamin B6, and vitamin B12 of the CUME project participants (*n* = 2695).

Folate		Vitamin B6		Vitamin B12	
Food	(%)	Food	(%)	Food	(%)
Beans/Lentil	23.45	Banana	11.08	Cheese	13.98
French Bread	10.01	Pork	5.14	Beef	11.97
Papaya	3.93	Beef	4.67	Fish	8.03
Brown bread	3.46	Loaf bread	4.65	Sardine/Tuna fish	6.85
Loaf bread	3.43	Beans/Lentil	4.35	Whole milk	6.66
Cheese bread	3.27	Cooked potato	4.22	Skimmed milk	6.14
Avocado	3.08	Natural juice	3.36	Egg	5.71
Banana	2.96	Orange/Tangerine	3.32	Salmon	4.46
Orange/Tangerine	2.79	Avocado	2.56	Fried snacks	4.28
Cress/Cabbage/Arugula/Spinach	2.57	Salmon	2.42	Nonfat yogurt	3.92
Sweet bread	2.05	Cassava/Yam	2.26	Yogurt	3.23
Egg	1.89	Beer	2.25	Viscera	2.75
Tomato	1.62	Brown bread	2.04	Semi-skimmed milk	2.74
Pizza	1.53	French Bread	2.01	Pork	2.74
Lettuce/Chard	1.43	Chocolate powder	1.91	Pizza	2.22
Chickpeas	1.40	Chili	1.86	Milk chocolate/Bonbon/Chocolate truffle	1.56
Guava	1.28	Skimmed milk	1.84	Franfurter/Sousage	1.58
Hot dog/Hamburger	1.19	Whole rice	1.71	Morning cereal	1.38
Morning cereal	1.17	Whole milk	1.57	Hot dog/Hamburger	1.29
Beet	1.16	Tomato	1.46	Ice cream	1.00
Cassava/Yam	1.16	Peanut/Chestnuts/Walnuts	1.38	-	-
Peanut/Chestnuts/Walnuts	1.10	Apple	1.32	-	-
Beer	1.04	Egg	1.31	-	-
-	-	Fish	1.24	-	-
-	-	Papaya	1.22	-	-
-	-	Pizza	1.11	-	-
-	-	Fried snacks	1.06	-	-
-	-	Lettuce/Chard	1.03	-	-
-	-	Morning cereal	1.00	-	-

**Table 3:** Prevalence ratio (PR) for excess body weight * and obesity ** according to dietary folate, vitamin B6, and vitamin B12 of the CUME participants (*n* = 2695).

	Model 1			Model 2		
	RP (IC95%)		*p* Value	RP (IC95%)		*p* Value
Excess body weight ^†^						
Folate (μg/d)						
T1 < 407.95	1.0			1.0		
T2 407.95–511.12	0.75 (0.67–0.84)		<0.001	0.77 (0.69–0.87)		<0.001
T3 > 511.12	0.76 (0.68–0.85)		<0.001	0.79 (0.71–0.89)		<0.001
Vitamin B6 (mg/d)						
T1 < 1.50	1.0			1.0		
T2 1.50–1.79	0.94 (0.84–1.05)		0.332	0.99 (0.89–1.12)		0.999
T3 > 1.79	0.88 (0.78–0.98)		0.032	0.91 (0.81–1.02)		0.112
Vitamin B12 (μg/d)						
T1 < 3.16	1.0			1.0		
T2 3.16–4.50	0.98 (0.88–1.11)		0.860	1.03 (0.91–1.16)		0.570
T3 > 4.50	1.00 (0.90–1.13)		0.878	1.05 (0.93–1.18)		0.385
Obesity ^†^						
Folate (μg/d)						
T1 < 407.95	1.0			1.0		
T2 407.95–511.12	0.57 (0.43–0.75)		<0.001	0.61 (0.46–0.81)		0.001
T3 > 511.12	0.58 (0.45–0.76)		<0.001	0.60 (0.45–0.78)		<0.001
Vitamin B6 (mg/d)						
T1 <1.50	1.0			1.0		
T2 1.50–1.79	0.88 (0.67–1.16)		0.382	1.07 (0.81–1.40)		0.608
T3 >1.79	0.75 (0.57–0.99)		0.043	0.86 (0.65–1.13)		0.286
Vitamin B12 (μg/d)						
T1 <3.16	1.0			1.0		
T2 3.16–4.50	1.04 (0.78–1.38)		0.761	1.25 (0.94–1.66)		0.122
T3 > 4.50	1.00 (0.75–1.32)		0.976	1.17 (0.88–1.55)		0.261

* Excess body weight: BMI ≥ 25 kg/m^2^ (WHO, 1998), BMI ≥ 28 kg/m^2^ (OPAS, 2002) for adults and elderly respectively. ** Obesity: BMI ≥ 30 kg/m^2^ (WHO, 1998; OPAS, 2002). ^†^ Sample represents 1028 participants with excess body weight and 271 with obesity. Poisson regression Model 1: adjusted for age and sex. Poisson regression Model 2: adjusted for sex (female/male), age (years), alcohol consumption (yes/no), physical activity (yes/no), smoking (never smoked/former smoker/currently smoke), television hours per day (>1 h/ ≤1 h), use of vitamin supplement (yes/no), and daily caloric intake.

**Table 4:** Prevalence ratio (PR) or odds ratio (OR) for excess body weight * and obesity ** for males and females according to tertiles of folate intake (μg/d) of the CUME project participants (*n* = 2695).

	Model 1			Model 2		
	RP (IC95%)		*p* Value	RP (IC95%)		*p* Value
Excess body weight ^†^						
Male						
T1 < 453.51	1.0			1.0		
T2 453.51–585.39	0.84 (0.72–0.98)		0.033	0.84 (0.71–0.98)		0.032
T3 > 585.39	0.83 (0.71–0.97)		0.024	0.84 (0.72–0.98)		0.036
Female						
T1 < 388.78	1.0			1.0		
T2 388.78–480.99	0.73 (0.62–0.85)		<0.001	0.78 (0.67–0.92)		0.003
T3 > 480.99	0.70 (0.60–0.82)		<0.001	0.74 (0.63–0.87)		<0.001
Obesity ^†^						
Male						
T1 < 453.51	1.0			1.0		
T2 453.51–585.39	0.58 (0.38–0.88)		0.012	0.56 (0.36–0.88)		0.012
T3 > 585.39	0.51 (0.33–0.80)		0.003	0.50 (0.32–0.79)		0.003
Female						
T1 < 388.78	1.0			1.0		
T2 388.78–480.99	0.65 (0.44–0.95)		0.030	0.78 (0.52–1.71)		0.234
T3 > 480.99	0.55 (0.36–0.82)		0.003	0.57 (0.37–0.86)		0.009

* Excess body weight: BMI ≥ 25 kg/m^2^ (WHO, 1998), BMI ≥ 28 kg/m^2^ (OPAS, 2002) for adults and elderly, respectively. ** Obesity: BMI ≥ 30 kg/m^2^ (WHO, 1998; OPAS, 2002). ^†^ Excess body weight sample composed of 431 people in the male group and 597 in the female group; for the sample with obesity, 106 in the male group and 165 in the female group. Poisson or logistic (obesity females) regression Model 1: adjusted for age (years). OR was performed for the female group due to a prevalence of obesity less than 10%. Poisson or logistic (obesity females) regression Model 2: adjusted for age (years), alcohol consumption (yes/no), physical activity (yes/no), smoking (never smoked/former smoker/currently smoke), television hours per day (>1 h/≤1 h), use of vitamin supplement (yes/no), and daily caloric intake. OR was performed for the female group due to a prevalence of obesity less than 10%.
